# Dose-dependent impact of enrofloxacin on broiler chicken gut resistome is mitigated by synbiotic application

**DOI:** 10.3389/fmicb.2022.869538

**Published:** 2022-08-04

**Authors:** Robin Temmerman, Mahdi Ghanbari, Gunther Antonissen, Gerd Schatzmayr, Luc Duchateau, Freddy Haesebrouck, An Garmyn, Mathias Devreese

**Affiliations:** ^1^Department of Pathobiology, Pharmacology and Zoological Medicine, Faculty of Veterinary Medicine, Ghent University, Merelbeke, Belgium; ^2^DSM - BIOMIN Research Center, Tulln, Austria; ^3^Faculty of Veterinary Medicine, Biometrics Research Center, Ghent University, Merelbeke, Belgium

**Keywords:** broiler chicken, enrofloxacin, gut resistome, gut microbiome, synbiotic

## Abstract

Fluoroquinolone agents are considered critical for human medicine by the World Health Organization (WHO). However, they are often used for the treatment of avian colibacillosis in poultry production, creating considerable concern regarding the potential spread of fluoroquinolone resistance genes from commensals to pathogens. Therefore, there is a need to understand the impact of fluoroquinolone application on the reservoir of ARGs in poultry gut and devise means to circumvent potential resistome expansion. Building upon a recent dose optimization effort, we used shotgun metagenomics to investigate the time-course change in the cecal microbiome and resistome of broiler chickens receiving an optimized dosage [12.5 mg/kg body weight (bw)/day], with or without synbiotic supplementation (PoultryStar^®^, BIOMIN GmbH), and a high dosage of enrofloxacin (50 mg/kg bw/day). Compared to the high dose treatment, the low (optimized) dose of enrofloxacin caused the most significant perturbations in the cecal microbiota and resistome of the broiler chickens, demonstrated by a lower cecal microbiota diversity while substantially increasing the antibiotic resistance genes (ARGs) resistome diversity. Withdrawal of antibiotics resulted in a pronounced reduction in ARG diversity. Chickens receiving the synbiotic treatment had the lowest diversity and number of enriched ARGs, suggesting an alleviating impact on the burden of the gut resistome. Some Proteobacteria were significantly increased in the cecal metagenome of chickens receiving enrofloxacin and showed a positive association with increased ARG burden. Differential abundance (DA) analysis revealed a significant increase in the abundance of ARGs encoding resistance to macrolides-lincosamides-streptogramins (MLS), aminoglycosides, and tetracyclines over the period of enrofloxacin application, with the optimized dosage application resulting in a twofold higher number of affected ARG compared to high dosage application. Our results provide novel insights into the dose-dependent effects of clinically important enrofloxacin application in shaping the broiler gut resistome, which was mitigated by a synbiotic application. The contribution to ameliorating the adverse effects of antimicrobial agents, that is, lowering the spread of antimicrobial resistance genes, on the poultry and potentially other livestock gastrointestinal microbiomes and resistomes merits further study.

## Introduction

Antimicrobial resistance (AMR) accounts for an annual death toll of approximately 700,000 people and is a major threat to global health (World Health Organisation (WHO), 2014). There is a general consensus that the alarming levels of AMR are strongly related to the overuse and misuse of antimicrobial agents in human and animal medicine, as well as in agriculture and livestock production systems (Anomaly, 2009; Chantziaras et al., 2014; Raboisson et al., 2020), where antimicrobial use is projected to increase further due to the growth of human and livestock populations (Van Boeckel et al., 2015). Fluoroquinolones are of critical importance in human medicine for the treatment of *Campylobacter*, *Salmonella*, and *Shigella* infections. These infections are zoonotic and limited therapeutic options are available (WHO Advisory Group on Integrated Surveillance of Antimicrobial Resistance (AGISAR), 2019). Due to their crucial status and the development of AMR in human medicine, fluoroquinolone use in the veterinary sector is under scrutiny. In the broiler industry, fluoroquinolone chemotherapeutics such as enrofloxacin are still frequently administered via drinking water for the treatment of colibacillosis (an infection that is caused by the enigmatic avian pathogenic *Escherichia coli*) in Europe and Asia (Persoons et al., 2012; Joosten et al., 2019; Roth et al., 2019b), which creates a considerable concern regarding the potential spread of fluoroquinolone resistance genes from commensal bacteria found in livestock to pathogenic organisms in humans (Dheilly et al., 2012; Gregova and Kmet, 2020; Temmerman et al., 2021). However, there is limited published information on the impact of enrofloxacin treatment on the poultry gut microbiome and, more importantly, no information on the development of resistome [the reservoir of antibiotic resistance genes (ARGs)] in poultry gut, leaving a considerable knowledge gap that limits the thorough evaluation and optimization of such critically important antibiotics in broiler production. In fact, the judicious use of antibiotics is extremely important to preserve the efficacy of antimicrobials while mitigating resistance development (World Health Organization (WHO), 2015; Foditsch et al., 2019). Using the 16S rRNA amplicon sequencing approach, previous studies have shown that treatment with enrofloxacin causes significant shifts in intestinal microbiota of chickens while hindering recovery from *Salmonella* Typhimurium and selecting for fluoroquinolone resistance in coliforms (Li et al., 2017a,b; Ma et al., 2020).

With regard to the use of enrofloxacin in the poultry sector, the European Medicines Agency’s Committee for Veterinary Medicinal Products urged to revise the currently approved dose of 10 mg/kg bw/day (European Medicines Agency, 2018, 2021), considering the development of resistance, as well as the notoriously large inter-animal variability in drinking behavior (Soraci et al., 2014; Ferran and Roques, 2019; Ferran et al., 2020), which can lead to both under- (treatment failure and resistance selection) and overexposure (adverse effects) in animals. In our recent efforts to optimize the dose of enrofloxacin for the treatment of colibacillosis in broiler chickens, using a novel drinking behavior pharmacokinetic (DBPK) model, we found a dosage of 12.5 mg/kg per day to be efficacious in treating colibacillosis caused by strains without acquired resistance mechanisms (Temmerman et al., 2021). However, *in vivo* evaluation of such optimized doses in broiler chickens is still lacking. The impact of other antimicrobials on the residential microbiota in broilers has been investigated (Xiong et al., 2018; Luiken et al., 2019; Turcotte et al., 2020), showing a range of effects, including reduction of overall diversity of the bacterial communities, microbiome metabolic shifts, increasing gut susceptibility to colonization, and stimulation of the development of AMR. In medical research, there are indications that alternatives to antibiotics, including dietary prebiotics, probiotics, and synbiotics (products that combine the simultaneous and potentially synergistic use of pre- and probiotics) (Gibson et al., 2004; Markowiak and Śliżewska, 2017) can reverse the perturbations induced by antimicrobial administration (Korpela et al., 2018; Abd El-Hack et al., 2020).

Building on our previous findings, we performed a shotgun metagenomics analysis to provide insights into the effects of enrofloxacin treatment on the broiler cecal microbiome and antimicrobial resistance gene (ARG) reservoir (resistome), applied at the optimized (12.5 mg/kg bw/day, OPT group) or high dose (50 mg/kg bw/day, HIGH group). Given the importance of studying the potential means to circumvent resistome expansion, we also evaluated the change in the cecal microbiome and resistome of broiler chickens supplemented with a synbiotic in conjunction with the administration of enrofloxacin (OPT_PS group). The findings of this study have important implications for poultry production and public health, as there is little research on the effects of therapeutic doses of enrofloxacin and synbiotics in conjugation with the application of enrofloxacin on the gut microbiome and resistome.

## Materials and methods

### Animals and experimental design

The animal trial was reviewed and approved by the ethical committee of the Faculty of Veterinary Medicine and Bioscience Engineering of Ghent University (EC 2020-016). The care and treatment of the birds were in full compliance with the national and European legislation concerning animal welfare (European Commission., 2010; Royal Decree., 2013). Throughout the study, feed and drinking water were provided *ad libitum*.

Eighty-four healthy 1-day-old male broiler chicks (Ross 308), with similar body weights (bws) of approximately 55 g were randomly allocated to one of four treatments [*n* = 7 cages (3 chickens each) per treatment]: (1) standard commercial feed (Table 1, Farm mash 1&2, Versele-Laga, Deinze, Belgium) for 37 days (control group), 2) antibiotic-treated group 1 [OPT, 12.5 mg/kg bw/day Baytril^®^ 10% oral solution (Bayer, Diegem, Belgium) consecutively via drinking water from day 21 to day 23 of age], (3) antibiotic-treated group 2 (HIGH, 50 mg/kg bw/day Baytril^®^ 10% oral solution consecutively via drinking water from day 21 to day 23 of age), and (4) synbiotic supplemented group (OPT_PS), in which chickens received the commercial feed supplemented with a synbiotic product (PoultryStar^®^, BIOMIN GmbH, Austria) during their lifetime, in conjunction with receiving enrofloxacin at a dose of 12.5 mg/kg bw/day via drinking water from day 21 to day 23 of age. The age of the birds was selected to reflect the real commercial setting and scenario for antimicrobial use, since colibacillosis frequently occurs in the second half of the broiler growing period. Additionally, the 3-day treatment duration was in compliance with the label of Baytril. The synbiotic supplement was composed of 2 × 10^8^ CFU/g multi-species probiotic: *Enterococcus faecium* (1.20 × 10^8^ CFU/g), *Bifidobacterium animalis* (6 × 10^7^ CFU/g), *Lactobacillus salivarius* (2 × 10^7^ CFU/g), and prebiotic inulin. The amount of mL enrofloxacin that needed to be added in the drinking water was calculated using the following formula (Equation 1) (Temmerman et al., 2021):


(1) Dose(m⁢gk⁢g)*averageBW(kg)*numberofanimalspergroup*a⁢m⁢o⁢u⁢n⁢t⁢o⁢f⁢w⁢a⁢t⁢e⁢r⁢(L)A⁢v⁢e⁢r⁢a⁢g⁢e⁢w⁢a⁢t⁢e⁢r⁢u⁢p⁢t⁢a⁢k⁢e⁢p⁢e⁢r⁢g⁢r⁢o⁢u⁢p⁢i⁢n⁢ 24⁢h⁢(L)

**TABLE 1:** Composition of the broiler diet (Farm mash 1&2, Versele-Laga).

Composition
Wheat, soya feed (produced from genetically modified soya), maize, wheat gluten feed, palm oil, calcium carbonate, sunflower seed feed, maize gluten feed, beet molasses, monocalcium phosphate, sodium chloride, sodium bicarbonate
**Analytical constituents**
Crude protein 18.0%, crude fat 5.0%, crude ash 5.5%, crude fiber 3.0%, lysine 0.97%, methionine 0.45%, calcium 0.95%, phosphorus 0.53%, sodium 0.10%
**Additives/kg**
*Nutritional additives*
3a672a vitamin A 10000 IU, 3a671 vitamin D3 2500 IU, 3a700 Vitamin E (all-rac-alpha-tocopheryl acetate) 80 mg, 3b103 iron (ferrous sulfate, monohydrate) 50 mg, 3b202 iodine (calcium iodate, anhydrous) 2.14 mg, 3b405 copper (cupric sulfate, pentahydrate) 10 mg, 3b502 manganese (manganous oxide) 75 mg, 3b603 zinc (zinc oxide) 70 mg, 3b802 selenium (coated granulated sodium selenite) 0.30 mg
*Zoo technical additives*
4a1617 endo-1,4-β-xylanase (EC 3.2.1.8) 1050 EPU, 4a16 6-phytase (EC 3.1.3.26) 250 FTU
*Technological additives*
Antioxidants
*Coccidiostats and histomonostats*
E763 lasalocid sodium 125 mg

### Cecal sampling, DNA extraction, library preparation and sequencing

Immediately before (day 20) and after enrofloxacin treatment (day 24), and 2 weeks after antibiotic withdrawal (day 37), one bird per replicate was sacrificed and digesta samples from the cecum were collected in Eppendorf cups, immediately snap frozen (liquid N2), and stored at −20°C. The animals were euthanized with pentobarbital IV (sodium pentobarbital 20%, Kela, Hoogstraten, Belgium) dosed at approximately 100 mg/kg.

DNA was extracted using the QIAamp PowerFecal Pro DNA Kit (Qiagen, Antwerp, Belgium), which as shown in our recent studies, has minimum effects on the microbial community structure and promising results in terms of the DNA integrity for chicken cecum samples (Wegl et al., 2021). The purity of the DNA was assessed by evaluating the 260/280 and 260/230 absorbances using NanoDrop^®^ (ND-1000, Thermo Fisher Scientific, Merelbeke, Belgium), while Quantus^®^ (Promega, Leiden, The Netherlands) was used to estimate the DNA concentration. Finally, DNA integrity was visually validated by gel electrophoresis. The DNA was sent to LGC Genomics GmbH, Berlin, Germany, where paired-end sequencing libraries were constructed using the Illumina Nextera XT Library Preparation Kit (Illumina Inc., San Diego, CA) followed by sequencing on the Illumina NextSeq 500 platform using high-output chemistry (2 × 150 bp) according to the manufacturer’s instructions.

### Sequenced data processing and statistical analysis

After demultiplexing and adaptor trimming (trimmomatic v. 0.39) (Bolger et al., 2014) taxonomic profiles of the demultiplexed reads and taxa relative abundance estimates were generated using Kraken2 (Wood et al., 2019) and Bracken (Lu et al., 2017), with kraken2 confidence set to 0.05 and bracken threshold to t −10. The abundances of ARGs were quantified by mapping the reads against the hand-curated AMR database MEGARes v2.0 (Doster et al., 2020) using USEARCH (v10) (Edgar, 2010). In MEGARes 2.0, the nodes of the acyclic hierarchical ontology included four antimicrobial compound types, 57 classes, 220 mechanisms of resistance, and 1,345 gene groups that classified the 7,868 accessions. High confidence matches to the sequence in the MEGARes database were obtained by considering the entire coverage of the query reads against ARG genes with an identity threshold of 90% (parameters were set as “-usearch-global -id 0.9, maxaccepts 1, threads 50”) (Ghanbari et al., 2019). The variance explained by each variable included in the trial was quantified by omnibus testing on Bray–Curtis dissimilarity matrices from metagenome data, using PERMANOVA with the adonis function in the R package Vegan (Dixon, 2003). Ordination was performed with log-transformed normalized reads on two dimensions with the “prcomp” ordinate function from the package “stats” v3.6.2 and visualized using the “fviz_pca” function from the “factoextra” function from the FaceFacefactoextra package v1.0.7.

Linear regression analysis was performed considering random differences between the treatment groups at the first sampling (for the control and enrofloxacin treatments) as covariate terms in the model if the richness (the number of unique taxa) and inverse Simpson diversity (the number and relative abundance of unique taxa) were affected by the different treatments. The Inverse Simpson diversity index was selected due to its biological interpretation aspect and since it does not tend to be as affected by sampling effort as other diversity indexes. The Mann–Whitney test was used for statistical comparisons between the pairs of groups at each sampling time point. Log-fold changes in abundance (of taxa and ARGs) between groups were determined by a negative binomial generalized linear model using DESeq2 version 1.26.0. (Love et al., 2014) in R, considering the statistical significance at BH-corrected *P* ≤ 0.10. In all statistical analyses, the cage was considered as the experimental unit.

## Results

### Synbiotic administration resulted in a significantly higher body weight in broiler chicken

**FIGURE 1:**
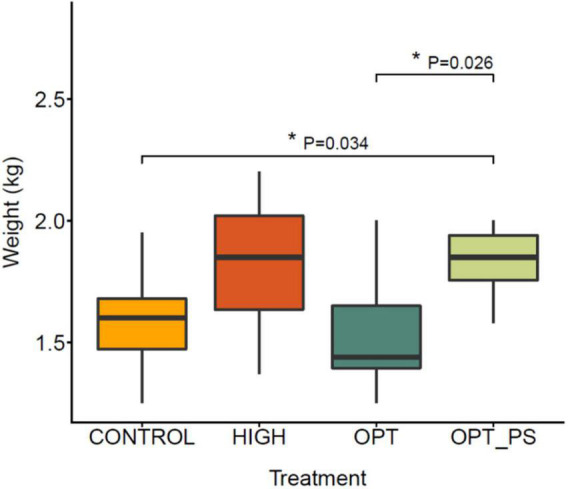
Box plot of the final body weight distribution of broiler chickens in different groups at 37 days of age. Control group (CONTROL); higher dose (HIGH): 50 mg/kg bw/day enrofloxacin; optimized dosing (OPT): 12.5 mg/kg bw/day enrofloxacin; optimized dosing with synbiotic supplementation (OPT_PS): 12.5 mg/kg bw/day enrofloxacin administration with synbiotic supplementation). FDR-corrected *p*-value from the Mann–Whitney statistical analysis is shown (*P < 0.05).

The average weights per treatment group (7 animals per group, 28 in total) and associated variability at the end of the animal trial are shown in Figure 1. The broiler weight ranges at day 37 were 1.25–1.95, 1.37–2.20, 1.25–2.00, and 1.58–2.00 kg for the control, HIGH, OPT, and OPT_PS groups, respectively. The corresponding averages are 1.59, 1.82, 1.54, and 1.83 kg, respectively. Chickens that received the highest enrofloxacin dose (HIGH group) and the optimized enrofloxacin dose supplemented with the synbiotic (OPT_PS group) had higher average weights at 37 days of age when compared to the control group, although only the chickens in the OPT with synbiotic supplementation (OPT_PS) group showed a much higher homogeneity in weight gain and a significant difference in weight group (*p* = 0.034, Figure 1). Interestingly, the difference between the optimized dosing (OPT) and OPT_PS was statistically significant (*p* = 0.026), suggesting an important role of synbiotic application in restoring physiological balance after perturbation by antibiotics and, consequently, improving the weight gain.

### Enrofloxacin and synbiotic applications were significantly associated with microbiota and resistome profiles variation in broiler chicken

**FIGURE 2:**
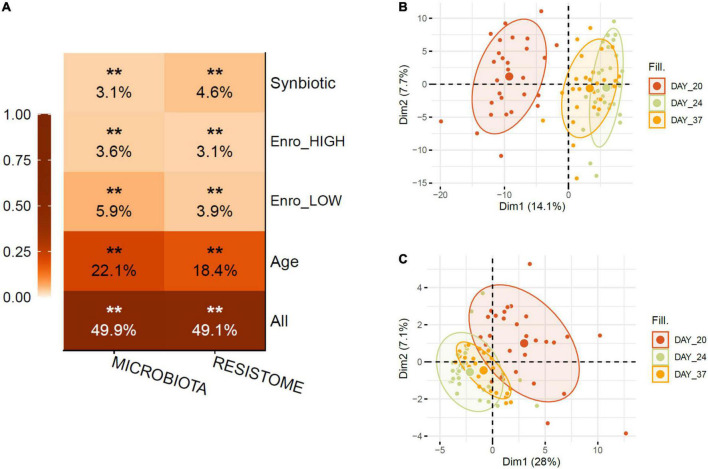
**(A)** PERMANOVA shows that the animal age had the largest effect on variation for both measurement types followed by the low/optimized dosage of enrofloxacin application. Stratified tests (Enro-HIGH/OPT (LOW), Synbiotic) consider only samples within the indicated category. Stars show FDR-corrected statistical significance (FDR **P ≤ 0.01). Variance is estimated for each feature independently, except for “All,” for which all metadata are included in the model. **(B)** Principle component analysis showing the changes in taxonomical and **(C)** ARG resistome profiles due to the effect of age. Ellipses indicate 95% confidence intervals of around centroids of the groupings with treatments as factor.

Shotgun metagenomics of the 84 cecum samples generated over 600 million paired-end sequence reads with a mean number of eight million reads per sample (see Supplementary File 1, which contains detailed information on the samples, including library size). On average, 23% of the reads per sample were classified to the species level using Kraken2 with a confidence threshold of 5%. Using the MEGARes v2.0 database with a 90% gene cut-off fraction, 1,281,069 reads were aligned to 1,000 antimicrobial resistance genes across treatments. Permutational Multivariate Analysis of Variance (PERMANOVA) statistical analysis calculated on Bray–Curtis dissimilarity matrices from metagenome data showed that the age of the birds (sampling time points: days 20, 24, and 37) was the largest driver of variation in both cecum microbiota and ARG resistome profiles, with antibiotic and synbiotic application capturing less variation (Figure 2A). In line with this, principal component analysis (PCA) revealed a clear separation of the sample based on the day of sampling (age) (Figures 2B,C). Regarding the effect of antibiotic administration on global diversity, the lower, optimized dosage of enrofloxacin (OPT) resulted in a higher variation compared to the higher dosage (HIGH) on the composition and structure of the taxonomical (Adonis R2, 5.9% vs. 3.6%) and the ARG resistome profiles (Adonis R2, 3.9% vs. 3.1%) in broiler chickens. Synbiotic application (OPT_PS) significantly explained 3.1 and 4.6% of the variation (Adonis R2) of the cecum microbiota and ARG resistome profiles, respectively (Figure 2A).

Further, pairwise *post hoc* comparisons^1^ showed a significant difference in the profiles of the relative abundance of taxa and ARGs (with the exception of the optimized dosage effect on the taxa profiles) between the enrofloxacin (both HIGH and OPT) and control treatment at day 24 of sampling. No significant differences were seen for the sample taken at day 20 or for those taken 2 weeks after the withdrawal of enrofloxacin at day 37 (Table 2), indicating the resilience of the bacterial communities carrying ARGs from antibiotic perturbation. On the other hand, OPT_PS showed a significant difference with the control groups in the relative abundance of taxonomy (with the exception of day 20) and resistome profiles at days 20 and 37 (Table 2). On day 24, immediately after an optimized dosage of enrofloxacin was applied in combination with the synbiotic product, the microbiota and ARG resistome profiles of the samples clearly diverged from the control group (*P* = 0,006), suggesting a synergistic effect on the chicken cecal metagenome (Table 2).

**TABLE 2:** Pairwise comparisons for permutational multivariate analysis of variance (PERMANOVA) using bray-cutis distance matrices.

		Sums of Sqs	Mean Sqs	F-Model	R2	*P*-value	*q*-value*
DAY_20CONTROL VS. DAY_20HIGH	Microbiota	0.041	0.041	0.794	0.062	0.583	0.631
	Resistome	0.012	0.012	0.589	0.047	0.905	0.905
DAY_20CONTROL VS. DAY_20OPT	Microbiota	0.094	0.094	1.508	0.112	0.127	0.186
	Resistome	0.023	0.023	1.265	0.095	0.239	0.258
DAY_20CONTROL VS. DAY_20OPT_PS	Microbiota	0.066	0.066	1.259	0.095	0.236	0.311
	Resistome	0.037	0.037	1.562	0.115	0.085	**0.098**
DAY_24CONTROL VS. DAY_24HIGH	Microbiota	0.109	0.109	2.598	0.178	0.005	**0.026**
	Resistome	0.043	0.043	3.988	0.249	0.005	**0.009**
DAY_24CONTROL VS. DAY_24OPT	Microbiota	0.087	0.087	1.456	0.108	0.146	0.209
	Resistome	0.034	0.034	2.277	0.159	0.003	**0.007**
DAY_24CONTROL VS. DAY_24OPT_PS	Microbiota	0.090	0.090	2.472	0.171	0.006	**0.026**
	Resistome	0.042	0.042	4.810	0.286	0.003	**0.007**
DAY_37CONTROL VS. DAY_37HIGH	Microbiota	0.032	0.032	0.707	0.056	0.740	0.763
	Resistome	0.031	0.031	1.639	0.120	0.090	0.101
DAY_37CONTROL VS. DAY_37OPT	Microbiota	0.028	0.028	0.569	0.045	0.897	0.897
	Resistome	0.014	0.014	1.024	0.079	0.444	0.457
DAY_37CONTROL VS. DAY_37OPT_PS	Microbiota	0.048	0.048	1.104	0.084	0.324	0.381
	Resistome	0.032	0.032	2.942	0.197	0.012	**0.017**

*FDR-corrected P-value. Bold values denote statistical significance.

### Enrofloxacin application resulted in a lower cecal microbiota diversity while substantially increasing antibiotic resistance gene resistome diversity

Linear regression analysis, taking into account the random differences between the high and optimized doses of enrofloxacin with control treatments on day 20, revealed a significant association of enrofloxacin application with the reduction of the microbiota Observed index from days 20 to day 24 (HIGH vs. control coefficient estimate day 24 = −41.143 with P = 2.55e^–05^, OPT vs. control coefficient estimate day 24 = −33.857 with *P* = 0.000414, and OPT_PS vs. control observed index coefficient estimate day 24 = −41.857 with *P* = 1.91e^–05^). Two weeks after the withdrawal of the antibiotic, although the microbial community richness recovered to some extent (HIGH vs. control CE day 37 = −26.429 with *P* = 0.00507, OPT vs. control EC day 37 = −26.000 with *P* = 0.005795, and OPT_PS vs. control CE day 37 = −20.143, with *P* = 0.030770), the cecum microbiota Observed index was still significantly lower in all groups than in the control. For OPT_PS at day 20, the observed index for the cecum microbiota was higher compared to those in the control (187 ± 9 vs. 183 ± 6), although the difference was not statistically significant.

**FIGURE 3:**
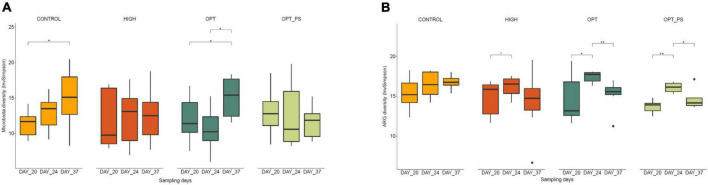
Diversity analysis (based on Inverse Simpson index) of the **(A)** microbial taxa and **(B)** ARGs in chicken cecal metagenome. FDR-corrected *p*-value from the Mann–Whitney statistical analysis is shown. *P < 0.05, **P < 0.01, respectively.

Calculation of the Inverse Simpson diversity index revealed that while the cecum microbiota diversity in the animals in the control group increased (not significantly) from day 20 (inverse Simpson 11.248 ± 1.819) to day 24 of age (12.842 ± 2.617), for the broiler chickens receiving the HIGH and OPT dosage, there was either no change (HIGH group, IS 12.048 ± 4.256–12.210 ± 4.0189) or a reduction (not significant; OPT group, IS 11.909 ± 3.059–10.536 ± 3.024) in microbial diversity (Figure 3A). For OPT_PS, the reduction in diversity was less pronounced (Figure 3A). At day 20 of age, OPT_PS showed generally higher diversity compared to the control (IS 12.960 ± 3.259 vs. 11.248 ± 1.819, respectively). While the withdrawal of enrofloxacin application did not seem to have a large impact on the recovery of the diversity for the animals in the HIGH and OPT_PS groups, it resulted in a significant increase in the cecum microbiota diversity in the optimized dosage group (Figure 3A).

A significant association of enrofloxacin application with the change in the diversity of ARGs was revealed [linear regression analysis, *F*_(11, 72)_ = 2.258, *p* = 0.02016 with an R2 of 0.26]. Over the course of the enrofloxacin application, that is, days 21–23, the diversity of ARGs in the cecum metagenome of the birds receiving enrofloxacin significantly increased (*P* < 0.05), regardless of dosage (Figure 3B). While OPT_PS had the lowest ARG diversity at day 20, the application of the optimized dosage of enrofloxacin on the top of the synbiotic supplement resulted in the largest increase (*P* < 0.01) on the diversity of ARGs from days 20 to 24 compared to all other treatments (Figure 3B). Yet, the cecum ARG diversity of birds in the OPT group (17.393 ± 0.727) at day 20 was significantly higher than that in the control (16.477 ± 1.651, *P* = 0.620), HIGH (16.176 ± 1.412, *P* = 0.097), and OPT_PS (16.089 ± 0.617, *P* = 0.011) groups, suggesting an alleviation effect of synbiotic application on the expansion of broiler cecal resistome (Figure 3B). Withdrawal of antibiotic application resulted in a pronounced reduction in ARG diversity, with OPT_PS showing significantly lower diversity of ARGs compared to the control group (*P* = 0.011) at day 37 (Figure 3B).

### Proteobacteria were significantly affected by the enrofloxacin application and are associated with an increased antibiotic resistance genes burden

**FIGURE 4:**
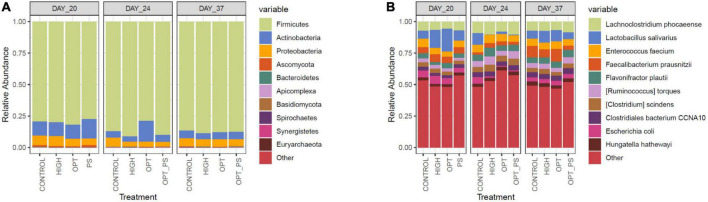
The 10 most abundant taxa in the bacterial communities at the phylum **(A)** and species **(B)** level, according to Kraken2 analysis.

Taxonomic profiling of the cecum samples in terms of the most abundant microbial phyla and species over the course of the study is shown in Figure 4. In total, 1,573 different microbial species were observed, of which species belonging to Firmicutes, followed by species belonging to Actinobacteria and Proteobacteria, were the most dominant members of the cecal microbiota (Figure 4).

**FIGURE 5:**
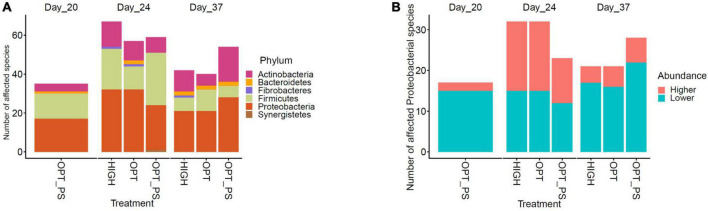
Impact of synbiotic and/or enrofloxacin application on differential abundances of microbiota. The bar plot shows the number of affected taxa **(A)** and Proteobacterial members (either increased or decreased) **(B)** based on differential abundance analysis in the chicken cecum.

Differential abundance (DA) analysis of the species-level taxonomic assignments using the DESeq2 tool revealed a significant effect of enrofloxacin application on shaping the cecal microbiota over the course of antibiotic application. Among the different groups of the impacted taxa, species belonging to the Proteobacteria phylum were among the most affected taxa due to enrofloxacin application followed by species from Firmicutes and Actinobacteria (Figure 5A and Supplementary Data 1). In fact, over the course of the enrofloxacin application (days 21–23), both high and optimized dose application resulted in a significant (FDR-corrected *p*-value < 10%) increase in the abundance of 17 proteobacterial species, while significantly reducing the abundance of 15 proteobacterial species (Figure 5B and Supplementary Data 1). Two weeks after the last day of antibiotic application, the number of affected microbial species belonging to the Proteobacteria decreased, with only four and five species showing a significantly higher abundance in the HIGH and OPT groups compared to the control, respectively (Figure 5B and Supplementary Data 1). Synbiotic application significantly (FDR-corrected *p*-value < 10%) reduced the abundance of 15 proteobacterial species, including *Escherichia coli* and *Campylobacter* spp., on day 20 (Supplementary Data 1). Interestingly, the OPT_PS group, showed a lower number of significantly increased Proteobacteria species compared to the OPT group (11 vs. 17, Figure 5B and Supplementary Data 1). Furthermore, with regard to the Firmicutes phylum, while species from the *Lactobacillus*, *Bacillus*, and *Anaerobutyricum* showed a significant increase (FDR-corrected *p*-value < 10%) in the cecum microbiota of chickens in OPT_PS compared to the control, several microbial pathogenic species such as *Enterococcus cecorum*, *Enterococcus hirae*, and *Enterococcus gallinarum* were significantly decreased (FDR-corrected *p*-value < 10%) (Supplementary Data 1). At day 37, 2 weeks after the withdrawal of antibiotics, the highest number of significantly decreased Proteobacteria was observed in the broiler chickens in OPT_PS (Figure 3).

**FIGURE 6:**
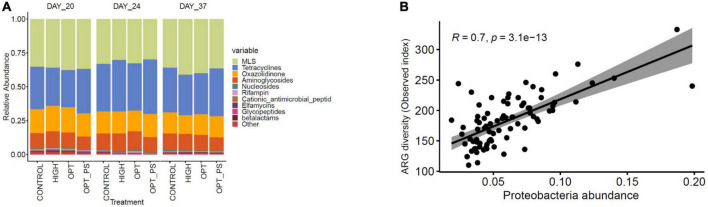
**(A)** Relative abundances of the top 10 classes of ARGs in the cecum digesta of medicated and non-medicated broiler chickens at different time points. **(B)** Scatterplots showing a strong Spearman’s rho correlation coefficient (R) between the ARG resistome load and the abundance of Proteobacteria in broiler chicken cecum metagenome samples.

**FIGURE 7:**
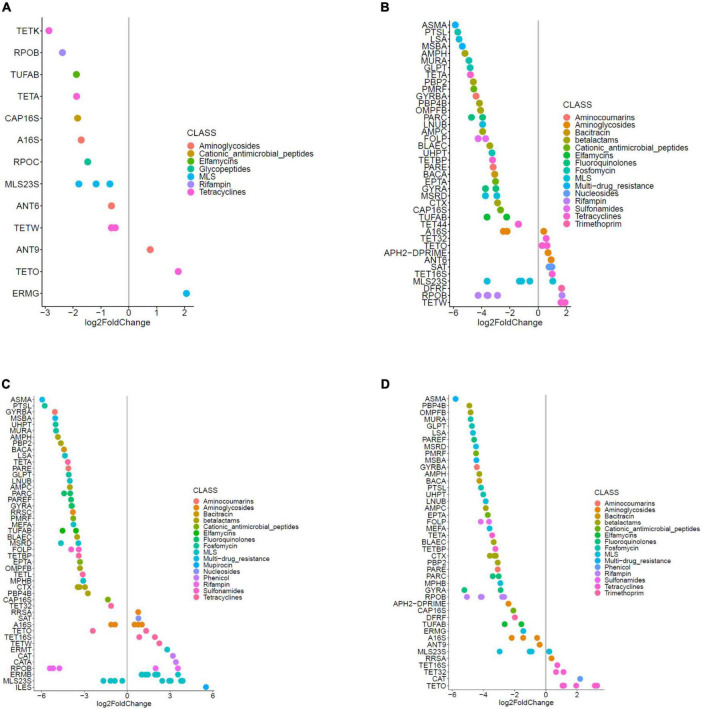
Significant (*q* < 0.10) log-fold changes in the abundances of ARG hits in **(A)** samples from OPT_PS taken at day 20, **(B)** samples from High group taken at day 24, **(C)** samples from OPT group taken at day 24, and **(D)** samples from OPT_PS group taken at day 24.

Antibiotic resistome profiling of the cecal metagenome showed that, regardless of antibiotic medication, all samples harbored a diverse range of ARGs (Figure 6A). Genes encoding MLS (mean = 29.64 ± 0.045 across all samples) and tetracycline (Mean = 27.70 ± 0,051 across all samples) resistance were the most predominant classes of ARGs to which the reads were aligned, followed by oxazolidinone and aminoglycoside resistance genes (Figure 6A). Correlation analysis showed that among the different dominant groups of the microbial phyla, Proteobacteria had the highest positive correlation (Spearman correlation, *R* = 0.7, *P* = 3.1e^–13^, Figure 6B) with the diversity of ARGs in the cecal metagenome of the broiler chickens (Figure 7B), with Actinobacteria showing a weak positive correlation (Spearman correlation *R* = 0.29, *P* = 0.0085) and Firmicutes showing a strong negative correlation (Spearman correlation *R* = −0.6, *P* = 1.2e^–10^).

**FIGURE 8:**
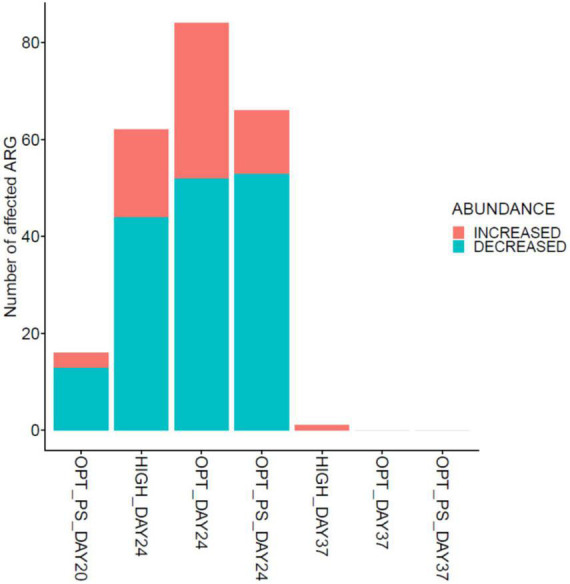
Impact of synbiotic and/or enrofloxacin application on differential abundances of ARGs. The bar plot shows the number of significantly affected ARGs (either increased or decreased) based on differential abundance analysis in broiler chicken cecum metagenomes.

In line with the observation that several members of Proteobacteria significantly increased in the cecal metagenome of broiler chickens receiving enrofloxacin, DA analysis revealed a significant increase in the abundance of ARGs encoding resistance to antibiotics belonging to different classes of antibiotics (mainly MLS, aminoglycosides, and tetracyclines) in these two groups from days 20 to 24, with the optimized dosage application resulting in a twofold higher number of DA ARGs compared to high dose application (32 DA ARGs vs. 17 DA ARGs, respectively; Figures 7, 8 and Supplementary Data 1). Examples of the increased ARGs in the samples from the HIGH and OPT treatments taken at day 24 include *cat* and *catA* (encoding chloramphenicol acetyltransferases), *ermB* (encoding 23S rRNA methyl-transferases), and MLS23S (macrolide-resistant 23S rRNA mutation) (Figures 7B,C). The most strongly diminished ARGs were the *asmA* (multi-drug RND efflux regulator) and *ptsL* (fosfomycin target mutation) genes, with an approximately −6 log2 fold change (Figures 7B,C). Other decreased genes included those encoding *gyrA*, *pbp2* (Penicillin binding protein2), and *CTX* (class A beta-lactamases), as well as *tetA*, *tetBP*, and *tet32* (Figures 7B,C). Withdrawal of antibiotic administration seems to result in the recovery of ARG resistome to the same level as the control group, as there were almost no DA ARGs in the cecum metagenomes of broiler chickens between the high/optimized dosage and those in the control group (Figure 8). For the samples taken from the OPT_PS group at day 20, where only synbiotic products were applied, the abundance of 13 ARGs was significantly lower than that in the control group, with three ARGs showing a significantly higher abundance in the chicken cecum metagenomes (Figures 7A, 8 and Supplementary Data 1). At day 24, for OPT_PS, an alleviating effect of synbiotic application on the ARG burden was observed (Figures 7D, 8 and Supplementary Data 1). Fifty-three ARGs (vs. 32 ARGs in OPT) were significantly lower in abundance in this group than in the control group, and only 13 genes (vs. 32 ARGs in OPT), mainly belonging to tetracycline resistance, showed significantly higher abundance compared to the control group (Figure 7D and Supplementary Data 1). No DA ARGs were found in the animals in this group at day 37 compared to the control group.

## Discussion

In this study, we characterized the impact of the critically important fluoroquinolone enrofloxacin following a lower, optimized (12.5 mg/kg bw/day) or high dosage (50 mg/kg bw/day) and its withdrawal on the broiler chicken cecal microbiota and associated resistome by applying shotgun metagenomics. In addition, as a unique aspect of our study, the effect of synbiotic administration in conjunction with the application of enrofloxacin on the cecal microbiota and its ARG reservoir was characterized.

Regarding the gallinaceous cecal microbiota, our study found Firmicutes abundance levels of approximately 80–90% on the three sample days (prior and post enrofloxacin treatment). This is in agreement with the previous observation that Firmicutes are the dominant group of microbial taxa in cecal microbiota over the 6-week lifespan of broilers (Oakley et al., 2014; Oakley and Kogut, 2016; Shang et al., 2018; Wegl et al., 2021). While Firmicutes bacteria are important members of both mammals and birds, there is inter-species variation. For example, the relative abundance of the Firmicutes phylum in the human cecum was comparable to that in broilers (Sun et al., 2019). However, in piglets, the dominance of Firmicutes was much less clear and was rivaled by the phylum Bacteriodetes (Ghanbari et al., 2019; Tan et al., 2019). Because of the similar basic composition of the cecal microbiota when compared with humans (Rychlik, 2020), the chicken cecum and residential microbiota can potentially function as a model for investigating microbiome and associated resistome dynamics in humans following different interventions, when validated with functional microbiome comparisons.

We report a significant dose-dependent impact of enrofloxacin application, with a high dose of the antibiotic causing a much higher perturbation of the diversity, structure, and composition of the cecal microbiota. This can be explained by the stronger bactericidal action of high-dose enrofloxacin compared to the lower dose. In line with this, it has been previously reported that enrofloxacin applied at a dose of 100 mg/kg bw completely eradicated *Salmonella* Typhimurium from chicken gut digesta immediately after administration (Li et al., 2017b) and was shown to have a significant effect on the chicken fecal and cecal microbiota structure evaluated by 16S amplicon sequencing in two independent studies (Li et al., 2017a; Ma et al., 2020).

Similar to previous observations in broiler chickens (Morales-Barrera et al., 2016; Li et al., 2017a; Ma et al., 2020), the Proteobacteria phylum was the most affected taxa by enrofloxacin application in the current study. The observed increase in the abundance of several Proteobacteria members in the medicated animals in the present study might be due to the potential of these taxa to carry ARGs. This is in accordance with previous reports that ARGs are more enriched in Proteobacteria and this phylum expands following antimicrobial treatment, thereby increasing the ARG burden (Young and Schmidt, 2004; Antonopoulos et al., 2009; Hu et al., 2016). Indeed, we observed a significant short-term expansion in the diversity and structure of ARG reservoirs in the cecum of the enrofloxacin-treated chickens, which was mitigated by the synbiotic supplementation in the group of chickens receiving the optimized dosage of enrofloxacin concomitantly with the synbiotic product. We believe that this beneficial effect can in fact be explained through ecological effects of the synbiotic supplement on the microbiome, such as its pronounced effect against strains that likely carry the expanded ARGs in the cecal microbiota. This is supported by the observation that, by day 20 of sampling, where all animals but those in the synbiotic group (OPT_PS) received no synbiotic supplement, a significant inhibition of several important pathobionts belonging to Proteobacteria, such as *E. coli* and *Campylobacter* spp., was observed in the cecal microbiota of the OPT_PS group. Furthermore, some other important pathobionts that may carry genes encoding acquired resistance to β-lactams and aminoglycoside antibiotics (Li et al., 2017a) and have potential negative impacts on chicken gut integrity, such as *Enterococcus cecorum*, *Enterococcus hirae*, and *Enterococcus gallinarum*, showed a lower abundance in the cecal microbiota of the OPT_PS group when they were receiving the optimized dosage of enrofloxacin (i.e., days 21–23) on the top of the synbiotic product. Such antagonistic effects have been previously observed and reported for the synbiotic product used in the current study (Ghareeb et al., 2012; Luoma et al., 2017; Roth et al., 2019a; Shanmugasundaram et al., 2019; Deng et al., 2020), which can explain the significantly higher bw gain of the OPT_PS group compared to the control. Interestingly, animals subjected to a HIGH dose of enrofloxacin (50 mg/kg bw/day) also achieved higher weights compared to the OPT dosage and control groups. Since fluoroquinolones are concentration-dependent antimicrobial agents (Levison and Levison, 2009), a possible explanation could be a stronger bactericidal action following the higher dose and subsequent larger shift in the microbiota, leading to a more pronounced growth promotion effect (Feighner and Dashkevicz, 1987; Butaye et al., 2003).

These findings raise the possibility that synbiotic-associated resistome expansion mitigation constitutes a unique observation stemming from our experimental design or the supplemented synbiotic product we utilized. Next, we aimed to determine whether other synbiotic studies reflected our findings. To our knowledge, there are no additional publicly available datasets displaying shotgun metagenomic data from the gastrointestinal microbiome of concomitantly synbiotic- and antibiotic-supplemented in either human or livestock species. However, in a recent study, probiotic colonization was associated with a reduced ARG load in endoscopic samples from 10 healthy individuals (Montassier et al., 2021). In contrast, the probiotic mix containing 11 strains from the *Lactobacillus*, *Bifidobacterium*, *Streptococcus*, and *Lactococcus* genera exacerbated the antibiotic-mediated resistome expansion in the lower gastrointestinal tract mucosa collected from 21 individuals. This observation may be due to the delay on microbiome recovery from antibiotics caused by probiotics, which consequently allowed for the expansion of species that likely carry the ARGs (Montassier et al., 2021). Additional studies are required to determine the effects of concomitant antibiotic and synbiotic application on the gut resistome.

Biodiversity analysis of the resistome results revealed the presence of a diverse range of resistance genes in the cecal microbiome of broiler chickens, even in the absence of antimicrobial pressure. However, there were substantial qualitative and quantitative differences between the cecal resistome investigated in this study and the results obtained from studies in pigs (Ghanbari et al., 2019), commercial feedlot cattle (Doster et al., 2018), poultry in other parts of the world (Guo et al., 2018), human feces (Pal et al., 2016), and environmental samples (Pal et al., 2016). This finding corroborates the assertion that ARGs are not randomly distributed in diverse environments but are the result of different selective pressures (Xiong et al., 2018; Ghanbari et al., 2019). The extent to which different enrofloxacin dosages affect resistance development in broiler chickens needs to be determined. We observed a detectable increase in the diversity and abundance of resistance genes, mainly macrolide resistance genes encoding rRNA methylases, followed by tetracycline resistance determinants in the cecum resistome of the chickens receiving the optimized dosage of enrofloxacin (i.e., 10 mg/kg bw/day), which was even higher than the observed increase (mainly tetracycline resistance determinants) in high-dose treated animals, although the gut resistome mainly recovered 2 weeks after cessation of treatment. Such collateral effects of antibiotic application (i.e., enrichment of ARGs not conferring resistance to the administered agent) have been observed before for fluoroquinolone (Sanders et al., 1984; Tolun et al., 2004; Macesic et al., 2014), oxytetracycline (Ghanbari et al., 2019), chlortetracycline, sulfamethazine, and penicillin (Looft et al., 2012) antibiotics, highlighting the need to carefully examine the patterns of multiple drug resistance that may occur simultaneously. While several enriched ARGs in the enrofloxacin-treated groups are based on resistance mutations of a chromosomal gene [e.g., A16S (Prammananan et al., 1998), MLS 23S rRNA (Sugiyama et al., 2017), *tet16S* (Ross et al., 1998)], the majority of enriched genes [*ermB* (Leclercq, 2002), *cat* (Shaw, 1983), *catA* (Shaw, 1983), *ileS* (Brown et al., 2003), *sat* (Shaw et al., 1993), *tetW/O/32* (Connell et al., 2003), *ant6* (Shaw et al., 1993), *aph2-prime* (Shaw et al., 1993), and *dfrF* (Gleckman et al., 1981)] are associated with mobile genetic elements, potentially explaining the observed collateral effects (Looft et al., 2012; Ghanbari et al., 2019). A high dose of enrofloxacin is likely to provide a sufficient concentration of the active drug in the intestinal contents. This potentially results in stronger bactericidal activity, effectively killing many (susceptible) commensals that likely harbor a variety of intrinsic and acquired ARGs. Consistent with our findings, three rounds of 7-day treatment alternated with 7-day withdrawal of enrofloxacin at a high dose (100 mg/kg bw) was effective in treating *Salmonella* Typhimurium infection while selecting for less resistance in both *Salmonella* Typhimurium and coliforms in chickens, compared to either PK/PD optimized (4 mg/kg bw) or 0.1 mg/kg/bw (Li et al., 2017b). Further trials and experiments are required to confirm these observations by looking beyond metagenomics-based resistome profiling i.e., at effects on ARG expression and by showing that enriched ARGs can be horizontally transferred to other commensals or pathogens and confer phenotypic resistance. In fact, a recently reported study showed that only around 60% of ARGs identified in broiler gut microbiota were expressed (Wang et al., 2020) when an integrated approach shotgun metagenomics and metatranscriptomics was used.

## Conclusion

In this study, we investigated the impact of synbiotics and clinically important fluoroquinolones on the cecal reservoir of ARGs in broiler chickens by applying a shotgun metagenomic sequencing approach. Our results provide novel insights into the dose-dependent effects of enrofloxacin application on shaping the broiler gut resistome, which was mitigated by a synbiotic application. The contribution to ameliorating the adverse effects of antibiotics, that is, lowering the spread of antimicrobial resistance genes, in poultry and other livestock gastrointestinal tracts merits further studies.

## Data availability statement

The datasets presented in this study can be found in online repositories. The names of the repository/repositories and accession number(s) can be found below: https://www.ncbi.nlm.nih.gov/, PRJNA761967.

## Ethics statement

The animal study was reviewed and approved by the Ethical Committee of the Faculty of Veterinary Medicine and Bioscience Engineering of Ghent University.

## Author contributions

RT, MG, GA, LD, and MD designed the study. MG, GA, GS, FH, AG, and MD supervised the experiments. RT, MG, and GA performed the experiments. RT and MG analyzed the results and wrote the manuscript. All authors read, revised, and approved the final manuscript.

## Footnotes

1
https://rdrr.io/github/GuillemSalazar/EcolUtils/man/adonis.pair.html
